# Commitment and Wellbeing: The Relationship Dilemma in a Two-Wave Study

**DOI:** 10.3389/fpsyg.2022.816240

**Published:** 2022-03-28

**Authors:** Maria José Chambel, Vânia Sofia Carvalho

**Affiliations:** Faculdade de Psicologia, Centro de Investigação em Ciência Psicológica, Universidade de Lisboa, Lisbon, Portugal

**Keywords:** commitment, burnout, engagement, health perceptions, two-wave

## Abstract

There has been little consensus around the sequential relationship between organizational affective commitment and workers’ wellbeing. In line with the Conservation of Resources Theory, results of this two-wave study with a contact center employee sample (*N* = 483) showed that organizational affective commitment decreases work ill-being (i.e., burnout) and increases work wellbeing (i.e., work-engagement). Furthermore, in keeping with the loss spiral assumption of this theory, the mediating role of burnout in the affective commitment-health relationship was supported in this study. However, in accordance with the Job Demand-Resources, work engagement was found not to prevent effects on health. The findings have implications for the organizational affective commitment theory, as well as for organizational occupational health policies and interventions.

## Introduction

Affective organizational commitment, referring to employees’ emotional ties to the company in which they work, has been highlighted as a necessary condition for companies’ performance effectiveness (e.g., Meyer et al., 2002; Grego-Planer, 2019). However, the effects of this attitude on employees, namely on their strain and wellbeing, have been under-studied and are therefore less understood (Meyer and Maltin, 2010). Given the acknowledged need for stress prevention and wellbeing promotion in both the United States and Europe (EU-OSHA, 2018; NIOSH, 2020), an understanding of their relationship with affective organizational commitment would aid managers to establish healthier workplaces (Guest, 2017).

In order to explain the relationship between affective organizational commitment and employees’ strain and wellbeing, two perspectives may be highlighted. The first, based on the Conservation of Resources Theory (COR, Hobfoll, 2002), assumes that it is this attitude that makes it possible to predict employees’ wellbeing. The commitment - (ill) wellbeing pathway may be theoretically explained by the assumption that resources – things people value centrally – should reverse a situation of strain characterized by resource loss, while organizational affective commitment may equip employees with the resources that enable them to cope with a loss resource situation and prevent the development of strain, thus ensuring their wellbeing (Meyer and Maltin, 2010). Therefore, according to this perspective, for an organization to guarantee the wellbeing at work of its employees, it should first focus on creating their affective tie toward the organization. By doing so, the organization effectively equips its employees with the resources they need to deal with the challenges presented to them by their work (Hobfoll et al., 2018). The second perspective, based on the Job-Demand Resources model (JD-R, Bakker and Demerouti, 2007), assumes that it is employees’ (ill) wellbeing at work that predicts their affective commitment to the organization. This model establishes that: in a motivational process, employees’ wellbeing (i.e., work engagement) has positive effects on positive organizational outcomes such as affective commitment (Schaufeli and Bakker, 2004); in a health impairment process, work strain (i.e., burnout) not only has associations with ill-health but also with positive organizational outcomes, such as affective commitment (Hakanen et al., 2008). Thus, according to this perspective, affective organizational commitment can only be ensured in a context where employees perceive wellbeing at work and not strain (Schaufeli and Bakker, 2004).

This study aims to clarify the sequential relationships between organizational affective commitment and wellbeing (i.e., wellbeing at work and context free wellbeing), examining the relationship between both. More specifically, it draws from the COR framework, which presupposes that strain results from a resource loss and wellbeing from a resource gain. It explores how organizational affective commitment may equip employees with resources and prevent the development of burnout (Meyer and Maltin, 2010) and promote the development of engagement (Hobfoll, 2002), which, in turn, promotes employees’ context free wellbeing. Conversely, based on the JD-R and the simultaneous motivational and health impairment processes, the premise that work engagement positively affects organizational affective commitment and burnout negatively affects organizational affective commitment and employees’ context free wellbeing is explored. This model was tested in a two-wave study conducted in a contact center.

This manuscript seeks to make a number of contributions. The first, namely a comparison of these two theoretical perspectives positing different relationships between wellbeing and commitment, is particularly informative as it determines the extent to which a dynamic relationship between employees’ wellbeing and organizational commitment is present (Abdelmoteleb, 2018). The second, from a practical viewpoint, contributes to more effective action on the part of management, namely human resources management in a contact center context. In the acknowledgment that this context favors stress and compromises wellbeing at work (Deery et al., 2002; Holman, 2002; Sprigg and Jackson, 2006; Castanheira and Chambel, 2010), if affective organizational commitment is an antecedent of wellbeing at work and strain, then the action adopted by the organization should be focused on the development of practices that foster this attitude. Conversely, if this affective tie to the organization is the result of stress and wellbeing then the organizational practices and actions should be focused on burnout prevention and engagement promotion.

From a methodological perspective, a two-wave study was performed to examine the relationship of these variables over time. Most prior studies on the relationship between commitment and employees’ wellbeing have used a cross-sectional design, and their ability to identify the direction of this relationship has thus been problematic (Meyer and Maltin, 2010; Abdelmoteleb, 2018). Finally, and in line with previous research, this study supports the assumption that strain and wellbeing at work (i.e., burnout, and work engagement) are predictors of context free wellbeing (i.e., health perceptions) (for a review see Bakker et al., 2014).

### Conceptual Framework and the Proposed Models

#### The Influence of Organizational Affective Commitment on Health: The Mediating Role of Burnout and Engagement

Burnout, which has a longer history, was identified in the 1970s by Freudenberger and Maslach as a state of emotional exhaustion and loss of motivation and involvement of professionals in their work context (Schaufeli and Enzmann, 1998). Burnout has been conceptualized as a crisis in the relationship with work, affecting employees of different professional areas, characterized by two core dimensions: exhaustion – general reactions to stress, such as emotional and physical fatigue, depression, psychosomatic complaints, and anxiety; cynicism (depersonalization) – an attitude of indifference or mental detachment and divestment toward work (Schaufeli and Buunk, 2003). Work engagement may be defined as a positive affective state at work, composed of vigor, dedication and absorption dimensions (Schaufeli et al., 2006). In more concrete terms, vigor is characterized by individuals’ ability to invest high levels of energy and mental endurance in their work activities, as well as the desire and ability to persist in overcoming difficulties in the work domain. Dedication is a feeling of enthusiasm, inspiration and pride toward the work domain. Absorption refers to individuals’ state of absolute concentration, reflecting their continuous involvement in work.

The Conservation of Resources (COR) theory has been used to explain the development of burnout and engagement (Hobfoll, 2002). A basic tenet of COR is that individuals desire to acquire and maintain valued resources, such as objects, personal characteristics, conditions, and energies that are crucial to gain and consolidate other resources. The threat of loss, actual loss or lack of a resource gain after investment are understood as demands that increase the likelihood of stress. Thus, as stress increases, individuals must progressively divert their psychological resources to combat its negative effects until those resources are depleted and they feel overwhelmed and no longer capable of coping with their work. Therefore, persistent experience of low resources and high demands is linked with deterioration of other valued resources, such as energy, identification, and perceived efficacy, which is the burnout process (Hobfoll et al., 1990). In fact, in the work context, burnout may be recurrent; principally as work demands usually involve a higher amount of resources used by employees, in comparison with the amount of resources they are able to replenish (Freedy and Hobfoll, 1994). Nevertheless, also in line with the COR, the relationship between job demands and strain (burnout) or wellbeing at work (engagement) varies in the investment of resources. In fact, this theory assumes that employees’ (ilI) wellbeing at work will increase or decrease over time in reaction to changes in resources, as is also the case with burnout, which will result from a “loss spiral,” and engagement from a “gain spiral.” High resources in a high demand setting should lead to optimal functioning, which in turn should foster a reinvestment of resources such as time and energy into the work setting. Therefore, the individual applies some resources in order to deal with threatening conditions and inhibit negative outcomes such as burnout (Hobfoll, 1989), but also uses these resources to gain other resources and stimulate positive outcomes, such as work engagement (Hobfoll, 2002).

Organizational affective commitment equips employees with resources that enable them to cope with high demand situations and prevent the development of burnout (Meyer and Maltin, 2010). Furthermore, these resources may be functional in achieving work goals, reducing job demands or stimulating personal growth and development, and also promote high work engagement (Schaufeli and Taris, 2014). Affective commitment implies that employees identify with the organization that provides them with feelings of support, control, resilience, sense of belonging and meaning and purpose, which help them cope with high demand situations (Greenaway et al., 2015; Steffens et al., 2017). Moreover, the involvement and affective liaison with the organization help employees perceive that they have access to more resources and, thus, they continue to devote effort and energy in situations with high demands (Tourigny et al., 2013). On the other hand, the positive emotions inherent to this affective commitment serve to broaden individuals’ momentary thought-action repertoires and build their enduring personal resources, including physical, intellectual, social, and psychological resources (Fredrickson, 2001; Rivkin et al., 2018). As highlighted by Fredrickson (2004), these personal resources act as reserves that can be used later to improve the odds of successful coping and survival, even in situations characterized by high demands. For example, affective organizational commitment may boost an individual’s self-esteem, which enables him/her to fulfill work obligations without overburdening personal resources, namely energy and time (Hobfoll, 2002).

Therefore, organizational affective commitment equips employees with resources that are central to survival or major goal attainment in their professional context (Hobfoll et al., 2018). In fact, this affective liaison with the organization may help employees efficiently manage stress and may give them more resilience, providing them with safety and protection against resource loss, promoting lower strain or burnout and providing them with the capacity and opportunity to obtain more resources that promote work wellbeing or engagement (Hobfoll et al., 2015).

The critical review conducted by Meyer and Maltin (2010) confirmed that a strong organizational affective commitment has benefits for employees’ wellbeing at work and context free wellbeing. However, empirical studies testing the effect of organizational commitment on burnout or on engagement are scarce. For both exceptions, in a cross-sectional study, Kalliath et al. (1998) observed that organizational commitment was negatively related to nurses’ burnout and to laboratory technicians’ exhaustion. Additionally, in a two-wave cross-lagged study, Yalabik et al. (2013) sampled clerical employees in the specialized lending division of a bank and found that affective commitment positively affected work engagement.

Moreover, existing research has consistently demonstrated positive relations between organizational affective commitment and different indices or indicators of health (Meyer and Maltin, 2010). Furthermore, despite their scarcity, some longitudinal studies have also confirmed the positive influence of organizational affective commitment on context-free wellbeing. Panaccio and Vandenberghe (2009) found that organizational commitment predicted employees’ context free wellbeing (i.e., positive and negative affect, and life satisfaction), while Abdelmoteleb (2018) confirmed that organizational affective commitment had a negative impact on job stress.

In line with the burnout literature (e.g., Melamed et al., 2006) and work engagement literature (e.g., Hakanen and Schaufeli, 2012), it is assumed that these work (ill) wellbeing indicators will influence employees’ health, and this process may also be understood through the Conservation of Resources Theory (COR; Hobfoll et al., 2018). As mentioned above, burnout will develop if coping is unsuccessful or if many resources are forced to be invested (Halbesleben et al., 2014). In fact, burnout results from long-term threats (e.g., excessively high job demands) to one’s energy resources and/or actual loss of these resources after heavily investing in work without appropriate gains in return (Hakanen et al., 2008). Hence, since burnout is associated with a progressive loss of resources, which undermines the individual’s coping abilities, this negative work-related state leads to a loss spiral that will spill over and generalize into negative general and context-free wellbeing (Hakanen and Schaufeli, 2012). Contrariwise, work engagement results from a positive spillover situation, as individuals who have resources are likely to gain more resources over time. In fact, individuals who have access to strong resource pools are more likely to seek opportunities to risk resources for increased resource gains (“gain spiral”) (Hobfoll and Shirom, 2000). Being engaged at work may therefore also spill over to context-free wellbeing by positively influencing health. This means that work engagement could offer protection against illness, as engagement is an active and energetic psychological state that fosters the mobilization of resources, even under mentally challenging conditions (Seppälä et al., 2012).

Different cross-sectional studies in a variety of contexts have pointed to burnout having a negative relationship with different context free wellbeing indicators, including happiness (Iverson et al., 1998) and satisfaction with life (González-Rico et al., 2016), and a positive relationship with health problems, namely psychosomatic health complaints (Schaufeli and Bakker, 2004) and ill-health (Hakanen et al., 2006). In the same vein, longitudinal studies have confirmed that burnout has a detrimental effect on life satisfaction (Hakanen and Schaufeli, 2012) and a positive influence on depressive symptoms (Hakanen et al., 2008; Hakanen and Schaufeli, 2012). Corroborating the prediction of health by work engagement, a previous cross-sectional study has shown that work engagement is positively related to healthy, adaptive cardiac autonomic activity, and especially increases parasympathetic activity (Seppälä et al., 2012). Similarly, Hakanen and Schaufeli (2012) verified that work engagement predicts life satisfaction over time.

Therefore, and for the purposes of this study, the COR (Hobfoll, 1989) is deemed to have a theoretical framework that offers a possible explanation for the influence of organizational affective commitment on wellbeing. First, as previously mentioned, employees with strong organizational affective commitment are either less likely to experience a resource loss or more likely to experience a resource gain, since they have greater access to resources to help them cope with the demands they encounter in their professional life and to obtain their goals and growth and development, which are consequently more protected from burnout and more eligible for work engagement. Second, this work (ill) wellbeing, namely (burnout) work engagement (prevents) contributes to the individual’s ability to cope with demands effectively, which may lead to a (loss) gain spiral and subsequently (deteriorate) promote his/her context-free wellbeing. Thus, (burnout) work engagement is expected to mediate the relationship between organizational affective commitment and employee health through the gradual (draining) gaining of resources that (burnout) work engagement represents. Based on the above, the following hypotheses are advanced:

Hypothesis 1: Burnout mediates the time-lagged relationship between organizational affective commitment and health perceptions.
Hypothesis 2: Work engagement mediates the time-lagged relationship between organizational affective commitment and health perceptions.

#### The Influence of Burnout and Engagement on Organizational Affective Commitment and Health

Alternatively, in line with the JD-R, wellbeing at work (i.e., burnout and work engagement) has effects on organizational affective commitment and health (Schaufeli and Bakker, 2004). This model assumes that job demands [i.e., those physical, psychological, social, or organizational aspects of the job that require physical, cognitive or emotional effort and are therefore associated with certain physiological and/or psychological costs (Demerouti et al., 2001, p. 501)] exhaust employees’ mental and physical resources [i.e., those physical, psychological, social, or organizational aspects of the job that may reduce job demands are functional in achieving work goals, and stimulate personal growth, learning, and development (Demerouti et al., 2001, p. 501)] and may therefore lead to burnout, which in turn leads to ill health and decreases affective commitment. On the other hand, job resources, promoting intrinsic or extrinsic motivation, enhance work engagement, which in turn increases affective commitment.

A cross-sectional study with teachers (Hakanen et al., 2006) confirmed the negative relationship between burnout and affective commitment, a positive relationship between burnout and ill-health and a positive relationship between work engagement and affective commitment. In the same vein, other cross-sectorial study found a negative association between burnout and commitment in nephrology nurses Li et al. (2021) and (Tuval et al., 2021) study burnout played a mediating role between job stress and organizational commitment.

Another multisource cross-sectional study also observed a positive relationship between work engagement and affective commitment (Boon and Kalshoven, 2014).

Further, other cross-sectorial study observed that work engagement plays a full mediation between job demand and organizational commitment (Khan et al., 2021). A cross-lagged study with dentists (Hakanen et al., 2008) confirmed that burnout had a positive cross-lagged effect on depression but not on organizational commitment, and work engagement had a positive cross-lagged impact on organizational commitment. Based on the above, the following hypotheses are advanced:

Hypothesis 3: Burnout has a negative time-lagged relationship with organizational affective commitment and with health perceptions.
Hypothesis 4: Work engagement has a positive time-lagged relationship with organizational affective commitment and with health perceptions.

**FIGURE 1:**
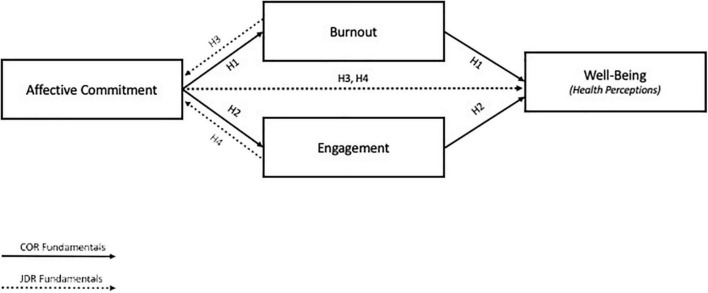
Theoretical model.

These two theoretical frameworks served as a backdrop to test the different models. Hypotheses 1 and 2 (Model 1) indicate that burnout and engagement partially mediate the effect of affective commitment on health, whereas Hypotheses 3 and 4 (Model 2) suggest the effect of burnout on health and affective commitment and the effect of engagement on affective commitment. Therefore, the four hypotheses together (Model 3) represent a reciprocal model in which there is a direct relationship between affective commitment and health. Moreover, burnout and engagement mediate the impact of these variables and have an effect on affective commitment. Figure 1 indicates the conceptual framework of the current study within a cross-lagged design context with two-waves.

## Materials and Methods

### Sample and Procedure

In May 2018, all the employees of a Portuguese contact center company (*N* = 1923), were invited by the Human Resource Manager to participate in a study on stress and wellbeing. The *survey monkey* platform was used to obtain the answers to the questionnaire and this platform allows absolute confidentiality because it allows not to collect the IP or Mac Address. An informed consent explaining the study goals and procedure was distributed to all employees of the contact center by Human Resource Manager department. As result, 1507 employees provided their informed consent (78.37%) completed the online questionnaire during working hours or at home. Anonymity was ensured and no incentive was offered for their participation. In May 2019, all the workers were again asked to respond to an identical questionnaire, and this time 1342 workers responded. At both points in time the questionnaire was available for one month and the employees received two reminders during that period. By means of a code created by each participant, it was possible to match the responses of each participant at both points in time. 483 (32,05%) valid responses were obtained for both waves of the study. Therefore, the final sample included 483 employees, of whom 333 were female (69.5%), in which the mean age was 33.24 years (SD = 7.75), where 63.4% had completed secondary school and 36,6% higher education. As far as tenure is concerned, 14.5% had been employed in the contact center from 3 to 6 months, 6.9% from 6 to 9 months, 9.4% from 9 months to one year, 41.3% from 1 to 5 years, and 27.9% from 6 to 10 years. Dropout analyses revealed no significant differences at T1 among any of the study variables between those who dropped out and those who did not.

### Measures

#### Affective Commitment

The affective component of commitment was assessed using a translation of the measurement tool proposed by Meyer et al. (1993), which had been previously used with Portuguese contact center employees (Chambel and Sobral, 2011; Geraldes et al., 2018). The scale consists of six items (e.g., “This organization has a great deal of personal meaning to me”), scored by respondents on a seven-point Likert scale ranging from “strongly disagree” (1) to “strongly agree” (7). Responses yielded good internal consistency (Cronbach’s alpha = 0.89 and.90 at T1 and T2, respectively).

#### Wellbeing at Work

##### Burnout

To measure this variable, namely through the main dimensions of exhaustion and cynicism, the Portuguese version of the “Maslach Burnout Inventory (MBI)-General Survey” (Maslach et al., 1996) was used. This measure consists of ten items, divided equally between the two dimensions and scored via a seven-point Likert scale, ranging from “Never” (1) to “Every day” (7). The exhaustion scale consists of five items (e.g., “I feel emotionally drained by my work”), as does the cynicism scale (e.g., “I doubt the value and usefulness of my work”). The exhaustion dimension presented a Cronbach’s alpha = 0.92 and 0.93 at T1 and T2, respectively. The measure was previously used with Portuguese contact center employees (Geraldes et al., 2018).

##### Work Engagement

Work engagement was measured using the shortened version of the Utrecht Work Engagement Scale (UWES- 9 items; Schaufeli et al., 2006). An item example for vigor is: “at my work, I feel bursting with energy”; for dedication: “I find the work that I do full of meaning and purpose,” and for absorption: “time flies when I am working.” The participants responded using a seven-point Likert scale, ranging from 1 (never/nothing) to 7 (always, every day). Responses yielded good internal consistency (Cronbach’s alpha = 0.95 and 0.95 at T1 and T2, respectively).

#### Context Free Wellbeing

##### Health Perceptions

The General Health Questionnaire (GHQ-12) (Goldberg and Williams, 1988) was used to measure health perceptions. The 12-item form of the GHQ has been shown to be a sensitive instrument within an organizational context (Banks et al., 1980). Items consist of a question asking whether the respondent has recently experienced a particular symptom or item of behavior rated on a four-point scale: (1) less than usual, (2) no more than usual, (3) rather more than usual, and (4) far more than usual. The scale consists of positively worded items (e.g., “Feel capable of making decisions about things?”) and negatively worded items (e.g., “Been feeling unhappy or depressed?), considered a unitary screening measure of general health. This scale was previously translated and validated for the Portuguese population (Laranjeira, 2008) and was also used previously with Portuguese contact center employees (Neto et al., 2018). Cronbach’s alpha was 0.91 and 0.90 for time 1 and time 2, respectively.

##### Control Variables

Gender and tenure were assumed to be associated either with commitment (Mathieu and Zajac, 1990) or with wellbeing (Schaufeli and Buunk, 2003). Accordingly, these variables were controlled, gender (by coding “0” if the respondent was male and “1” if the respondent was female) and tenure (1 = less than 3 months; 2 = between 3 and 6 months; 3 = between 6 and 9 months; 4 = between 9 and 12 months; 5 = between 1 and 5 years; 6 = between 5 and 10 years; and 7 = more than 10 years).

### Analytical Method

The analyses were conducted by using Mplus 7.4 (Mutheìn and Mutheìn, 2015), with the robust maximum likelihood estimator (MLR). Numerous fit indices were employed, including the chi-square difference tests, the comparative fit index (CFI), the root mean square error of approximation (RMSEA) and the standardized root mean square residual (SRMR). For CFI, values greater than 0.90 represent a good model fit, and for SRMR and RMSEA, values below 0.07 indicate a good model fit (Arbuckle, 2008).

Initially, the suitability of the measurement models underlying the responses to the various instruments was verified, based on the recommendations of Pitts et al. (1996). Their longitudinal measurement invariance was analyzed across the time points to ascertain the extent to which a certain construct remained the same over time and in terms of stability, i.e., the degree to which the relative ordering of subjects remained the same over time. Accordingly, a nested measurement model in which the factor loadings of all the relevant constructs were restricted to be equal across the two waves was compared with a measurement model in which these factor loadings were set free. The chi-square difference statistic was used to determine the extent to which this assumption held true.

Furthermore, to control for the common method variance, our structural model was compared with a one-factor model (in which all items loaded onto a single latent variable). Additionally, since the data were from a single-sourced and self-administrated questionnaire, there is a possibility for common method variance (CMV) and thereby examined the CMV. Initially, as suggested by Podsakoff et al. (2003) procedural remedies were followed in the data collection process. For robustness, the most popular Harman’s one-factor test was executed.

The mean scores of the variables were then computed and paired sample *t*-tests were performed to examine the evolution over time.

**FIGURE 2:**
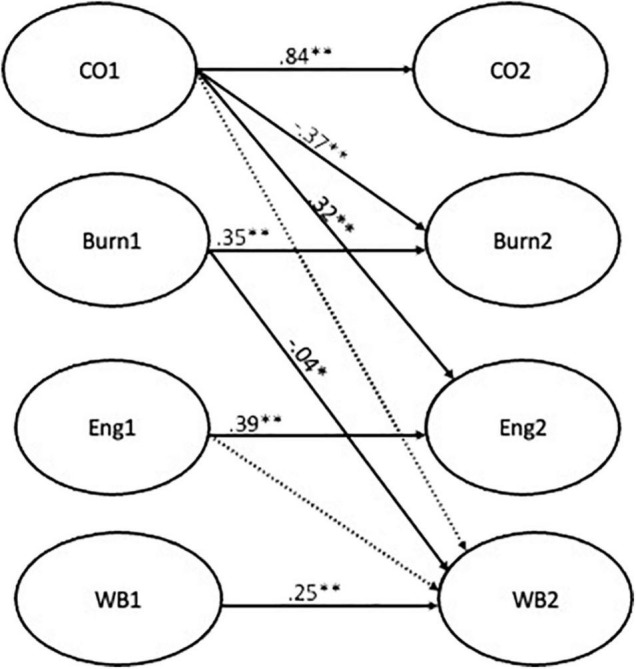
Result of model 1. **p < 0.01; *p < 0.05.

**FIGURE 3:**
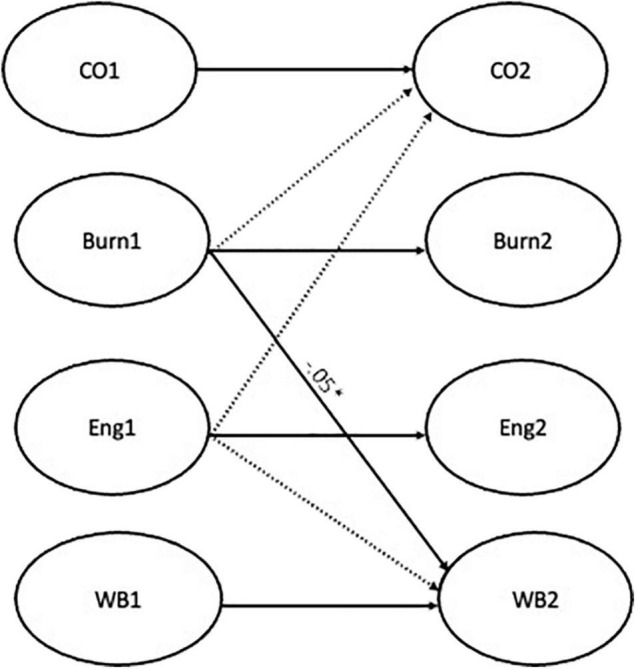
Result of model 2. *p < 0.05.

**FIGURE 4:**
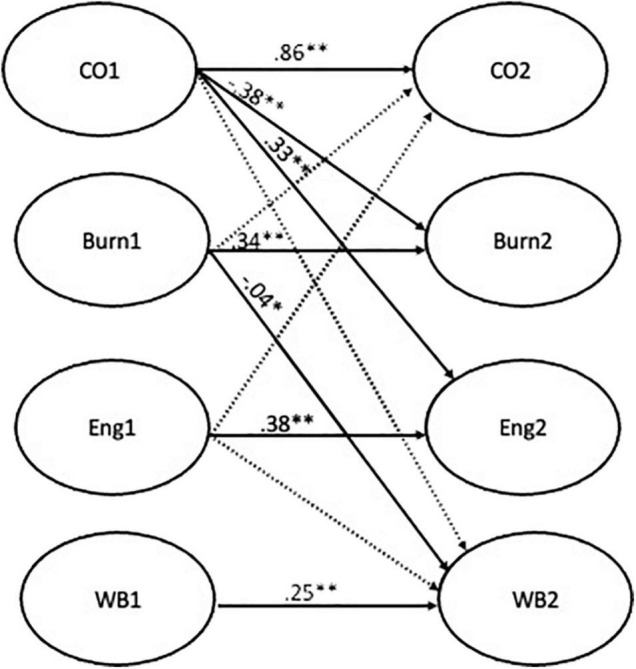
Result of model 3. **p < 0.01; *p < 0.05.

In a second phase, the research questions were analyzed using autoregressive cross-lagged paths. Auto-regression effects were included to control for the baseline levels of each latent variable (Gollob and Reichardt, 1991), synchronous correlations between the latent variables were allowed, and the error terms of each observed variable at T1 were allowed to co-vary with the corresponding indicator at T2, as is usually performed in longitudinal structural models. All the models included stability coefficients between commitment, burnout, engagement, and health at both measurement times. The first model, testing hypothesis 1 and 2, was performed for both mediators (i.e., burnout and engagement) and presented cross-lagged paths from commitment (time 1) to health perceptions (time 2), from commitment (time 1) to burnout and engagement (time 2) and, lastly, from burnout (time 1) and from engagement (time 1) to health perceptions (time 2) (Figure 2). The second model Figure 3, testing hypotheses 3 and 4, included a cross-lagged path between burnout/engagement (time 1) to affective commitment (time 2) and a path between burnout/engagement (time 1) and health perceptions (time 2). A third model integrating the paths from model 1 and model 2 was performed (Figure 4). The magnitude of indirect effects was calculated, and their significance was tested. For this process the “MODEL INDIRECT” command of Mplus software was used, which estimates and tests specific indirect effects (MacKinnon, 2008) and bootstrapping for bias-corrected indirect effects estimation was also included.

## Results

Measurement models were constructed, including the four distinct constructs (burnout, represented as a second-order variable consisting of exhaustion and cynicism, engagement, affective commitment and health perceptions). The fit indices for the measurement model (see Table 1) with free-factor loadings across the two points of time, showed acceptable results, χ2 (2550) = 5566.35, *p* < 0.01; CFI = 0.90; RMSEA = 0.05, SRMR = 0.05. Next, one-factor model was performed. Compared the one-factor model with the measurement model, the measurement model fit the data much better, leading to the assumption that a one-factor model could encompass the four constructs for each point of time, Δχ^2^ (31) = 7416.73, *p* < 0.01. These results confirmed the construct validity of the measurement model. Furthermore, the model in which the factor loadings for each construct of the four variables were limited to be equal across the two points of time was nested within the measurement model with free-factor loadings. The model with equal factor loadings showed acceptable results (χ^2^ (2576) = 5961.26, CFI = 0.90; RMSEA = 0.05, SRMR = 0.08, *p* < 0.01) and differed significantly in fit, compared with the model with the free-factor loadings [Δχ^2^(26) = 394.91, *p* < 0.01]. Accordingly, the model with equal factor loadings was preferred over the model with free-factor loadings, supporting the measurement invariability of the measured constructs.

**TABLE 1:** Testing construct validity and longitudinal constraints of the measurements’ models.

Model	χ2	DF	CFI	RMSEA	SRMR
One-factor model	12983.08**	2581	0.65	0.09	0.10
Four-factor model with free factor loadings	5566.35**	2550	0.90	0.05	0.05
Four-factor model with equal factor loadings	5961.26**	2576	0.01	0.05	0.08

**p < 0.01.

Moreover, the results show no indication of CMV since the variance for the time 1 model was 24% and for time 2 model was 22%, that is, less than 50%.

Paired sample *t*-tests demonstrated mean-level changes in the variables between T1 and T2. More specifically, affective commitment was found to be significantly lower at T2 compared to T1: *t*(483) = 61.54, *p* < 0.001; work engagement levels decreased from T1 to T2: *t*(483) = 59.44, *p* < 0.01; and health perceptions were significantly poorer: *t*(383) = 89.48, *p* < 0.001.

As may be seen in Table 2, the correlation matrix revealed that the constructs were practically significantly correlated with large effects to their corresponding homologs over time. This signified that the constructs remained stable over time, which was important for the design purposes of the present study. Moreover, the correlations between each pair of variables were also observed to be in the expected directions and of meaningful magnitude. Furthermore, gender and tenure reveal significant correlations with study variables reinforcing the need to control these variables.

**TABLE 2:** Means, Standard Deviations (SD) and Correlations among study variables (*n* = 483).

	Mean	SD	Gend_t1	Gend_t2	Ten_t1	Ten_t2	AC_t1	AC_t2	Ex _t1	Ex _t2	Cyn_t1	Cyn_t2	Eng_t1	Eng_t2	HP_t1
Gend_t1	_____	____													
Gend_t2	_____	____	1**												
Ten_t1	4.58	1.43	–0.03	–0.03											
Ten_t2	5.42	0.49	−0.10*	−0.10*	0.62**										
AC _t1	4.44	1.39	0.06	0.06	−0.22**	−0.26**									
AC_t2	4.05	1.45	0.06	0.05	−0.16**	−0.18**	0.64**								
Ex_t1	3.79	1.68	0.10*	0.10*	0.24**	0.21**	−0.50**	−0.35**							
Ex_t2	4.27	1.68	0.13*	13*	0.02	0.05	−0.34**	−0.44**	0.50**						
Cyn_t1	2.79	1.50	0.02	0.02	0.25**	0.19**	−0.51**	−0.40**	0.71**	0.34**					
Cyn_t2	3.10	1.54	0.07	0.07	0.05	0.06	−0.37**	−0.47**	0.40**	0.69**	0.43**				
Eng_t1	4.68	1.55	–0.01	–0.01	−0.27*	−0.32*	0.67**	0.51**	−0.54**	−0.35**	−0.54**	−0.37**			
Eng_T2	4.26	1.60	–0.02	–0.02	−0.14**	−0.16*	0.48**	0.62**	−0.42**	−0.57**	−0.43**	−0.55**	56**		
HP_T1	2.26	0.55	–0.03	–0.03	–0.08	–0.02	0.03	0.00	0.04	–0.01	–0.01	0.04	0.02	0.02	
HP_T2	2.11	0.52	−0.12*	−0.12*	–0.03	−0.11*	0.21**	0.29**	−0.24**	−0.47**	−0.23**	−0.36**	0.17**	0.40**	0.03

**p < 0.01; *p < 0.05. Gen, gender; Ten, tenure; AC, affective commitment; Ex, exhaustion; Cyn, cynicism; Eng, engagement; HP, health perceptions. Gender was a dummy variable (0, men; 1, women); tenure was an ordinal variable (1, less than 3 months; 2, between 3 and 6 months; 3, between 6 and 9 months; 4, between 9 and 12 months; 5, between 1 and 5 years; 6, between 5 and 10 years; 7, more than 10 years).

The results of the structural equation models (Table 3) showed that Model 1 fit the data well [χ^2^(2842) = 6011.84, *p* < 0.01, CFI = 0.90, RMSEA = 0.05, SRMR = 0.05]. The results showed that employees’ affective commitment (time 1) negatively predicted burnout and positively predicted engagement (time 2) (β = −0.36, *p* < 0.01 [−0.52, −0.20]; β = 0.30, *p* < 0.01 [0.18,0.43], respectively). Additionally, burnout (time 1) negatively predicted employees’ health perceptions (time 2) (β = −0.05, *p* < 0.05, [−0.08, −01.]), but the same was not observed for engagement (time 1) in relation to employees’ health perceptions (time 2), which presented a non-significant cross-lagged path. The relationship between employees’ affective commitment (time 1) with employees’ health perceptions (time 2) was non-significant. Furthermore, the indirect effect of commitment (time 1) on health perceptions (time 2) through the burnout (time 2) was positive and significant (β = 0.04, *p* < 0.05, [0.01,0.07]). As such, our first hypothesis was supported, and our second hypothesis was refuted.

**TABLE 3:** Structural equation models.

Models	χ2	DF	CFI	RMSEA	SRMR
Model 1	6011.84**	2842	0.90	0.05	0.05
Model 2	5795.38**	2838	0.90	0.05	0.06
Model 3	6011.17**	2840	0.90	0.05	0.05

**p < 0.01.

Regarding Model 2, a good fit was also observed [χ^2^(2838) = 5795.38, *p* < 0.01, CFI = 0.90, RMSEA = 0.05, SRMR = 0.06]. However, the path from burnout (time 1) to health perceptions (time 2) (β = −0.05, *p* < 0.05, [−0.08, −0.01]) was found to be the unique significant cross-lagged path. Hence, our third hypothesis was partially supported, and our fourth hypothesis was refuted.

Our reciprocal model, Model 3, presented a good fit [χ^2^(2840) = 6011.17, *p* < 0.01, CFI = 0.90, RMSEA = 0.05, SRMR = 0.05]. In terms of cross-lagged paths, employees’ affective commitment (time 1) was observed to negatively predict burnout and positively predict engagement (time 2) (β = −0.36, *p* < 0.01 [−0.53, −0.21]; β = 0.31, *p* < 0.01 [0.19,0.46], respectively). Moreover, burnout (time 1) negatively predicted employees’ health perceptions (time 2) (β = −0.05, *p* < 0.01, [−0.09, −0.01]). The cross-lagged paths between burnout (time 1) and affective commitment (time 2), between engagement (time 1) and affective commitment (time 2) and health perceptions (time 2), as well as between affective commitment (time 1) and health perceptions (time 2) were all non-significant. The indirect effect of commitment (time 1) on health perceptions (time 2) through the burnout (time 2) was non-significant. Lastly, Model 3 was compared with Model 1 [Δχ2 (2) = 0,67, non-significant] and Model 3 with Model 2 [Δχ2 (2) = 215.79, p < 0.01]. This enabled us to conclude that the COR framework was more relevant to the data.

Concerning the control variables, it is important to note that tenure was significantly and negatively related with time 1 commitment [β = −0.18, *p* < 0.01, (−0.25, −0.12)], burnout [β = −0.13, *p* < 0.01, (−0.09, −0.01)] and health perceptions [β = −0.08, *p* < 0.01, (0.08, 21)] and with time 2 burnout [β = −0.35, *p* < 0.01, (−0.57, −0.12)]. Gender was significantly and positively correlated with time 1 burnout [β = 0.29, *p* < 0.01, (0.07,0.47)] and health perceptions. [β = −0.08, *p* < 0.01, (−0.16, −0.02)].

## Discussion

According to the COR, organizational affective commitment affects employees’ (ill) wellbeing and, by contrast, according to the JD-R model, employees’ (ill) wellbeing affects organizational affective commitment. This study sought to test both these alternative models of the organizational commitment-(ill) wellbeing relationship. To this end, in line with the COR, an initial model was developed in which there was a direct relationship between organizational affective commitment and health perceptions, mediated by work (ill) wellbeing (i.e., burnout and work engagement). Alternatively, in line with the JD-R, a second model was developed, in which there was a direct relationship between work (ill) wellbeing and affective commitment and health. Finally, a third model integrating both these models was developed. Methodologically, the prior cross-sectional studies that have dominated the literature on the relationship between organizational commitment and wellbeing have limitations (Meyer and Maltin, 2010). Therefore, a two-wave cross-lagged study was conducted to test these models. The first proposed model fit the data well. Moreover, this model was favored over the second model which posited that work (ill) wellbeing was an antecedent of organizational affective commitment and health, and over the third model integrating the proposed paths of both models.

By testing alternative theories to explain results theory development is promoted (Leavitt et al., 2010). Accordingly, this study enhances the theory on the effects of organizational affective commitment on wellbeing (i.e., wellbeing at work and context free wellbeing), showing the contribution of the COR by testing it against other competing theory-driven models. This study confirmed that organizational affective commitment decreases work ill-being (i.e., burnout) and increases work wellbeing (i.e., work-engagement), thus supporting the argument that this positive emotional liaison equips employees with important resources that lead not only to the prevention of resource loss but also to resource gain (Hobfoll et al., 2018). These observations are in line with previous studies reporting the negative relationship between commitment and strain at work (Lambert et al., 2008; Abdelmoteleb, 2018), namely burnout (Kalliath et al., 1998), and the positive relationships between commitment and wellbeing at work, namely work engagement (Yalabik et al., 2013). However, the focus of these prior studies was to investigate only one indicator of work strain or one indicator of work wellbeing while this study has broadened its scope to integrate the COR principles of resource loss and gain processes and addresses both the negative and positive indicators of employee wellbeing. More specifically, the identification, involvement, and positive emotional liaison that affective commitment assumes may give employees more resilience, providing them with safety and protection against resource loss or threat of resource loss and promoting resource gain and growth (Hobfoll et al., 2015). Nevertheless, research including both the positive and negative indicators of wellbeing at work has not been conducted to date, and this gap may limit theoretical progress. As such, our findings provide more insights into the dual impact affective commitment may have on work ill-being and on work wellbeing.

Another interesting finding presented by this study is the mediating role of burnout in the affective commitment-health relationship. Essentially, support for this mediation is in line with the COR assumption of loss spiral, which establishes that burnout results from a resource loss that hinders the employee’s ability to confront his/her demands that will spill over and generalize into negative general and context-free wellbeing (Hakanen and Schaufeli, 2012). However, the assumption that work engagement mediates the relationship between affective commitment and health was not supported by this study. Unlike the COR gain spiral assumption, which establishes that this positive psychological state in work enhances the employee’s ability to gain more resources that will spill over into positive general and context-free wellbeing, in this study, work engagement did not affect health. This result, particularly the distinctive effect of burnout and work engagement on health, is in line with the JD-R, which establishes that in the health-impairment process, job strain (i.e., exhaustion, burnout) has negative effects on health, but in a motivational process work engagement has effects on positive organizational results (i.e., absence frequency, extra role performance) but does not prevent effects on health (Bakker and Demerouti, 2017). The two-wave study of Hakanen et al. (2008) confirmed this assumption and observed that burnout compromised future health (i.e., increases depressive symptoms) while work engagement had no effects on this indicator of ill-health. Moreover, this result is also in line with the first principle of COR that defends that resource loss is disproportionality more salient than recourse gain (Hobfoll et al., 2018) since with the same time length we verify that burnout negatively affect health perceptions but engagement has no significant effects in health.

Therefore, our study supported the COR assumption that organizational affective commitment predicted work (ill) wellbeing, namely negatively predicting burnout and positively predicting work engagement. However, in line with the JD-R, our study demonstrated findings similar to those from other studies (Bakker et al., 2014), pointing to burnout having a negative effect on health but work engagement not showing an effect on this indicator of context-free wellbeing.

### Limitations and Future Research

This study has several limitations. The first issue is the two-wave cross-lagged study which, despite its advantages over cross-sectional studies, cannot be considered longitudinal as it is not sufficiently informative with regard to the direction of influence among the study variables (Ployhart and Vandenberg, 2010). The development of future studies that include three or more waves could increase the reliability of the obtained results, namely the mutual reciprocity between variables and the inclusion of a mediator between them. Additionally, and considering the dynamic relationship between organizational affective commitment and wellbeing across time, studies need to be conducted in the future with more complex designs which quantify time-series data with repeated measurements of these variables and analyze the non-linear relationships between them (Navarro et al., 2020). The second issue involves the common method bias, due to the use of self-reports to collect the data. However, opting for a two-wave design is expected to mitigate the potential impact of this bias (Podsakoff et al., 2003). Furthermore, self-reported methods are valid to measure employees’ attitudes, perceptions and wellbeing (Spector, 2006). Notwithstanding, future studies that include objective health measures could be informative. The third issue is the arbitrary selection of the study time intervals. Selig and Preacher (2009) noted that although there is no theoretical or empirical basis for choosing the length of intervals between measurement occasions, the time lag should be long enough for the change process to have time to unfold. Given this understanding, a one-year lag was selected as an appropriate time interval for this study to analyze the change of employees’ attitudes and wellbeing. Future longitudinal studies with different and more varied time lags are necessary to draw more solid conclusions with regard to the dynamic between commitment and wellbeing. Furthermore, tenure and gender were included as control variables and presented significant paths with the main variable’s studies. As such, future studies should explore the moderating role of these variables. Finally, should also be taken in consideration that this study was realized with one occupational sector particularly acknowledged by high stress levels, i.e., call-centers As such, future studies should invest in different occupational sectors to inspect the generality of the results.

### Practical Implications

The findings of this study have important implications for practitioners. This study found a negative relationship between organizational affective commitment and burnout, a positive relationship between this positive attitude and work engagement and an indirect relationship between this positive attitude and health through burnout. Organizational affective commitment appears to be crucial to ensure employees’ wellbeing, not only in a work context but also in context-free conditions. Thus, highlighting actions that increase affective commitment is a valid way of promoting wellbeing. For example, as shown by Malhotra et al. (2007), promotional opportunities in a contact center context were significantly and positively related to employee affective commitment. On the other hand, in a recent multilevel study, Alcover et al. (2020) found that the level of performance-contingent rewards (team-level) guides the team’s autonomous motivation (team-level) which, in turn, fosters employees’ affective commitment (individual-level). With an exchange characterized by an economic transaction – the level of performance-contingent rewards – the contact center contributed to a social relationship with its employees and this relationship was positively related to their affective commitment. In high-turnover work environments, such as contact centers, and in the face of demand for increasingly competent and skilled employees, these human resources management practices that promotes organizational affective commitment can be highly effective not only in facilitating organizational retention policies and even increasing their attractiveness to qualified future employees, but also in the construction of a healthy context that will ensure employees’ wellbeing.

## Data Availability Statement

The raw data supporting the conclusions of this article will be made available by the authors, without undue reservation.

## Author Contributions

MC was involved in the conceptualization, data collection, original draft preparation, and writing of the manuscript. VC was involved methodology, data analysis, and review and editing process. Both authors contributed to the article and approved the submitted version.

## Conflict of Interest

The authors declare that the research was conducted in the absence of any commercial or financial relationships that could be construed as a potential conflict of interest.

## Publisher’s Note

All claims expressed in this article are solely those of the authors and do not necessarily represent those of their affiliated organizations, or those of the publisher, the editors and the reviewers. Any product that may be evaluated in this article, or claim that may be made by its manufacturer, is not guaranteed or endorsed by the publisher.
